# Hyaluronic acid and chondroitin sulfate, alone or in combination, efficiently counteract induced bladder cell damage and inflammation

**DOI:** 10.1371/journal.pone.0218475

**Published:** 2019-06-25

**Authors:** Antonietta Stellavato, Anna Virginia Adriana Pirozzi, Paola Diana, Sabrina Reale, Valentina Vassallo, Alessandra Fusco, Giovanna Donnarumma, Mario De Rosa, Chiara Schiraldi

**Affiliations:** 1 Department of Experimental Medicine, Section of Biotechnology, Medical Histology and Molecular Biology, Università della Campania “Luigi Vanvitelli”, Naples, Italy; 2 Department of Experimental Medicine, Section of Microbiology and Clinical Microbiology, Università della Campania “Luigi Vanvitelli”, Naples, Italy; University of Patras, GREECE

## Abstract

Interstitial cystitis and/or bladder pain syndrome (IC/BPS) are characterized by discomfort, abdominal pain, and pelvic pain, and they are often associated with chronic diseases. Pathological conditions related to IC/BPS can occur due to a defect in the integrity of the bladder lining. This defect has been ascribed to damage to the glycosaminoglycan (GAG) layer of the urinary epithelium. In addition, the incipient cascade of inflammation events might prompt extracellular matrix degradation. Several medical devices based on GAG instillation were proposed to re-establish epithelial integrity by GAGs binding to proteoglycans or interacting with structural urothelium. However, to date, only *in vitro* studies have investigated the GAG, hyaluronic acid (HA). In the present study, TNFα treatment was used to mimic IC/BPS-induced damage in bladder cells in an *in vitro* model. Highly purified fermentative HA and pharmaceutical grade bovine chondroitin sulfate (CSb), alone or in combination, were evaluated for the ability to counteract bladder cell damage. We evaluated NF-κB with western blots, and we analyzed interleukin 6 and 8 expression at the transcriptional and protein levels with quantitative RT-PCR, western blotting, and ELISA. We also evaluated the expression of an antibacterial peptide, human β-defensin-2. We confirmed our results in a 3D bladder epithelium model. Our results demonstrated that inflammatory status was reduced in the presence of HA, CSb, and the combination of both (HA/CSb 1.6%/2% w/v). This result suggested that these GAGs might be suitable for treating IC/BPS. All the assayed biomarkers showed that HA/CSb treatment modulated cells towards a more physiological status. Finally, we compared two commercial products suggested for the IC/BPS treatments and found that the product with more Ca^++^, showed enhanced anti-inflammatory activity and provided superior mucoadhesivity.

## Introduction

Bladder epithelium is a specialized tissue, which is protected by glycosaminoglycans (GAGs), like hyaluronic acid (HA) and chondroitin sulfate (CS) [1]. In chronic inflammatory bladder diseases and in different types of cystitis, GAGs are lost from the bladder lining [2]. Interstitial cystitis/bladder pain syndrome (IC/BPS) is a clinical condition characterized by discomfort or pain in the bladder and in the surrounding pelvic region associated with increased urinary urgency and frequency. This condition negatively affects the patient’s quality of life. Persistent loss of extracellular matrix GAGs might be responsible for inflammation, which results in chronic bladder epithelial damage [3]. The exact etiology of interstitial cystitis is unknown. However, based on the hypothesis that damage to the GAG layer is among the main causes of IC/BPS symptoms, intravesical GAG replenishment therapy has been widely used to treat patients that do not respond to conservative or oral therapy [4]. To administer intravescical GAGs, a catheter is introduced into the bladder and a highly viscous liquid is slowly instilled to allow it adhere to the bladder epithelium and exert the desired biological functions, which leads to symptom improvement.

Currently, natural heteropolysaccharides appear to be preferred in formulations on the market, and replacement therapy with different GAGs is recommended in a number of current clinical guidelines for IC/BPS treatment [5]. In particular, solutions containing: heparin, hyaluronic acid (HA), chondroitin sulfate (CS), and combination formulations of HA and CS, comprise a minimally-invasive alternative for treating patients with IC/BPS without adverse effects [5].

**Fig 1:**
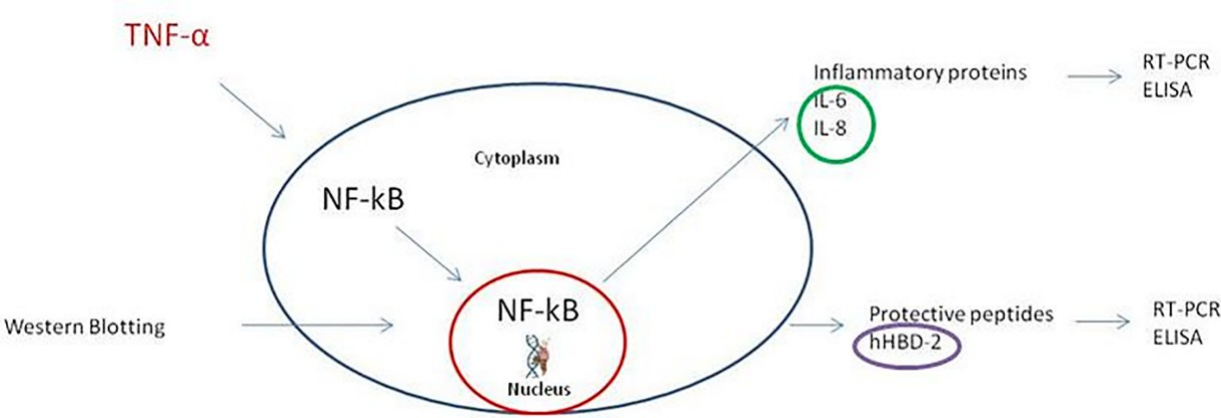
Schematic drawing of the signaling pathway related to the inflammation response to a TNFα insult, in the *in vitro* IC/BPS model.

IC/BPS induces a strong inflammatory response, starting with an injury to the epithelial barrier. In fact, several cytokines were upregulated in bladder tissues of patients with IC/BPS. Rooney and collaborators [6] used an *in vitro* model, based on urothelial cells pre-insulted with TNFα, to evaluate the effects of treatments with HA (0.4 mg/mL) on inflammatory biomarkers [6] (Fig 1).

HA and CS are currently included in some commercial formulations for treating IC/BPS symptoms. Thus, to unravel the key mechanisms at the basis of product functionality, in the present study, we investigated the efficacy of highly pure HA and pharmaceutical grade CS, alone or in combination, and at different concentrations, on the previously characterized *in vitro* IC/BPS model of urothelial cells pre-insulted with TNFα [6]. In addition to this 2D model, we also investigated a recently available 3D bladder epithelium model, which might provide a better representation of real tissue characteristics. We analyzed the inflammatory profile of this *in vitro* IC/BPS model. Specifically, we evaluated interleukin 6 (IL-6) and interleukin 8 (IL-8) expression, which are modulated by NF-κB-mediated signaling. Moreover, we reasoned that the loss of GAGs from the bladder wall lining could lead to increased trans-urothelial permeability [6]. Accordingly, we used immunofluorescence staining to evaluate the expression of the tight junction protein, zonulin (ZO-1), one of the key regulators of permeability. In addition, we studied the ability of bladder cells to secrete host defense peptides, like human beta defensin-2 (hBD-2) when incubated in media supplemented with HA and CS. In fact, defensin secretion may counteract microbial pathogen infections [7]. Finally, we compared two commercially available intravesical instillation products (IALURIL IBSA, Switzerland and INSTILLAMED FarcoPharma, Germany), which contain HA and CS and are used to treat IC/BPS. We tested their mucoadhesivity in the 2D biological model adopted for this study.

## Materials and methods

### Materials

The raw materials used in this study were: highly purified linear hyaluronan (SHyalt, Batch code 100004368) obtained from Altergon Spa (Italy) and pharmaceutical grade chondroitin sulfate (CSb code 8088, lot n° 15/0002), extracted from bovine cartilage and donated by IBSA SA (Switzerland). The single powders were used to prepare a combination formulation (HA/CSb) after thermal treatment in an autoclave. IALURIL samples were donated from IBSA SA. This medical device consisted of 50-mL pre-filled syringes that contained 800 mg HA (1.6% w/v), 1000 mg CS (2% w/v) and 440 mg calcium chloride (0.88% w/v). We also purchased 50-mL syringes pre-filled with INSTILLAMED (FarcoPharma, Germany), which contained 800 mg HA (1.6% w/v) and 1000 mg CS (2% w/v). Mucin (from porcine stomach, type II, cat. N. M2378) and Na_3_PO_4_ (cat. N. 342483) were purchased from Sigma-Aldrich (Milan, Italy). Phosphate-buffered saline (PBS) and fetal bovine serum (FBS) were purchased from GIBCO (Milan, Italy). All PCR reagents were obtained from Biorad Laboratories, (Milan Italy). All other reagents were obtained from Sigma-Aldrich (Milan, Italy), unless stated otherwise.

### Methods

#### Hydrodynamic characterization

We performed hydrodynamic analyses of the raw HA and CSb materials (Table 1) and their combined formulation. These reagents were prepared for biological experiments with size exclusion chromatography coupled with a triple detection array (SEC–TDA; Viscotek Malvern Instruments, UK). Details on the system and the analytical conditions were reported previously [8, 9].

**Table 1:** Molecular weights (MW), dispersity (Mw/Mn), intrinsic viscosity (IV), and recovery, based on the refractive index (RI), of HA, CSb, and HA/CSb formulations after thermal treatment.

Samples	MW (kDa)	Mw/Mn	IV (dL/g)	RI recovery (%)
HA	473 ± 15.65	1.85 ± 0.03	10.92 ± 0.20	95.60 ± 030
CSb	16.58 ± 0.14	1.21 ± 0.02	0.47 ± 0.05	101.04 ± 0.10
HA/CSb (1.6%/2.0 w/v)	432 ± 23.33/ 16.40 ± 0.31	1.35 ± 0.13/ 0.38 ± 0.03	9.59 ± 0.25/ 0.46 ± 0.02	98.2 ± 0.42

Data are the average of triplicate experiments. Abbreviations: HA: hyaluronic acid; CSb: bovine chondroitin sulfate; HA/CSb: mixture of HA and CSb.

#### Dynamic viscosity and mucoadhesive properties

Dynamic viscosity and mucoadhesion were measured with a rotational rheometerPhysica MCR 301 (Anton Paar, Germany), equipped with a coaxial cylinder (CC27-SN7969), a measuring cup diameter/measuring bob diameter ratio of 1.0847 (according to ISO 3219: gap length 39.984 mm, sample volume 19.00 mL), and a Peltier temperature controller.

The dynamic viscosity (η) of the samples was recorded as a function of the shear rate (0.01–1000 s^−1^), measured at 25°C, for HA, CSb, and the HA/CSb formulation, and at 37°C, for the IALURIL and INSTILLAMED products. We used 50 measuring points and no time setting. From each flow curve, we obtained the value of zero-shear viscosity (η_0_, viscosity in the range of a Newtonian plateau). The mucoadhesivity was evaluated as previously described with slight modifications [10; 11; 12; 13]. Briefly, the following samples were prepared for each determination:

1. a suspension of mucin (10 w/v%) buffered with a saturated solution of Na_3_PO_4_;
2. a sample solution diluted 1:1.8 with 0.15 M NaCl;
3. a suspension containing mucin (10 w/v%) and the sample under investigation at the concentration prepared in sample 2.

At each shear rate, the mucoadhesiveness of sample 2 was expressed as:

Δ(%)=[ηmuc+HA−(ηmuc+ηHA)]/(ηmuc+ηHA)×100;

where Δ(%) is the mucoadhesion index, ηmuc is the dynamic viscosity of sample 1, ηHA is the dynamic viscosity of sample 2, and ηmuc+HA is the dynamic viscosity of sample 3. For mucoadhesive polymers, the dynamic viscosity of sample 3 (ηmuc+HA) is expected to be higher than the sum of the viscosities ηmuc and ηHA, separately measured, due to the interaction between the polymer and mucin. The difference in percentage, Δ(%), is a measure of mucoadhesion strength [11,12,13].

Samples 1 and 3 were prepared as follows: Sample 1: mucin was hydrated with sterile water (10 h, 300 rpm, room temperature) to a final concentration of 25 w/v%. The pH of the resulting suspension was 3.8–4.0. Then, a saturated solution of Na_3_PO_4_ was added to bring the pH to 7.0–7.6. Water was added to achieve a final mucin concentration of 10 w/v%. Sample 3: a 25 w/v% mucin suspension was buffered with a saturated solution of Na_3_PO_4_. Then, the formulation to be tested was added to achieve the same sample and mucin concentrations found in samples 2 and 1, respectively. The final pH values were 6.5–7.1, except for IALURIL (pH 5.2), and the final conductivity values were 12.0–16.0 mS/cm, for all samples. Any variations in pH or conductivity within these ranges were not expected to affect viscosity.

#### Elemental analyses of commercial products

Sodium and calcium (in mg/kg) were quantified by applying an in-house Inductively Coupled Plasma-Optical Emission Spectroscopy (ICP-OES) method suitable for several kind of matrices. Duplicate commercial samples of IALURIL (lot 180505) and INSTILLAMED (lot LK04331) were diluted with an aqueous acidic solution and analyzed with ICP-OES (Agilent 720, Santa Clara, CA).

#### Cell culture and a 3D bladder epithelium model

A primary human bladder cell line (H-BLAK) was obtained from CELLnTEC Advanced Cell Systems AG (Bern, Switzerland). Cells were maintained at 37°C in a humidified atmosphere containing CO_2_ (5% v/v) in CnT Prime medium. The 3D human bladder epithelium preparation (RT112) and the relevant culture medium were obtained from Sterlab (Vallauris Cedex, France). The epithelium was cultured in standard medium, according to the manufacturer’s suggestion, and maintained for five days in an incubator with a humidified atmosphere (95% air/5% CO_2_ v/v) at 37°C to obtain the same characteristics as those observed *in vivo*.

#### TNF-α treatment to induce an *in vitro* inflammatory response

To simulate the pathology of IC/BPS, cell monolayers and human bladder epithelium were pre-incubated with TNF-α (10 ng·mL^-1^). The positive control contained TNF-α alone. Test samples contained different test solutions, including: HA (0.6 and 1% w/v), CSb (0.6 and 2% w/v), and a formulation of HA combined with CSb (1.6% w/v HA+2% w/v CSb) diluted (1:3) to achieve a final concentration of 0.53% w/v HA and 0.66% w/v CSb in culture. All experiments were performed with and without TNF-α treatment. Experiments that involved h-BD2 expression were performed without inducing inflammation.

#### Gene expression analyses

After all treatments, we evaluated IL-6 and IL-8 expression as inflammatory markers [6]. Briefly, RNA was extracted from cells, and reverse transcription was performed to obtain cDNA. cDNA samples were amplified with qRT-PCR, according to protocols designed for specific primers. Data analyses were performed with the 2^-^ΔΔ^Ct^ method to determine relative expression with respect to the control sample and a housekeeping gene (the internal control). The full PCR protocol was reported previously [14, 15].

#### NF-κB levels

We quantified NF-κB protein levels, because NF-κB is known to mediate inflammation. Briefly, we extracted cell proteins with RIPA lysis buffer (Amersham Biosciences, GE, USA). Protein concentrations were determined with the Bio-Rad protein assay reagent (Bio-Rad Laboratories, Milan, Italy). Equal amounts of protein (10 μg) were separated with SDS-PAGE and transferred to a nitrocellulose membrane. The membranes were incubated with rabbit anti-NF-κB antibodies (1:200 dilution; Santa Cruz Biotechnology, Santa Cruz CA) and goat anti-actin antibodies (1:500 dilution; Santa Cruz Biotechnology, Santa Cruz CA) at room temperature for 2h. Membranes were washed three times for 10 min and incubated for 1 h with 1:10000 dilutions of horseradish peroxidase (HRP)-conjugated anti-rabbit and anti-goat secondary antibodies (Santa Cruz Biotechnology, Santa Cruz, CA). Blots were developed with the ECL system (Amersham Biosciences, GE, USA) according to the manufacturer’s protocols. The full experimental procedure was described previously [16].

#### Secreted IL-6, IL-8, and hBD-2 concentrations

After stimulating bladder cell cultures with HA, CSb, or HA/CSb formulations, we quantified IL-6, IL-8 [6, 17], and hBD-2 [7, 18] protein levels with ELISA kits, according to the manufacturer’s instructions (Boster antibody and ELISA experts, Tema Ricerca, Italy). Briefly, cell media were removed from the wells and centrifuged, and the supernatants were transferred, to microtiter plates for incubation(in duplicate) with specific biotinylated polyclonal antibodies. Next, the mixtures were washed with PBS or TBS buffer. Then, an avidin-biotin-HRP complex was added, and unbound conjugates were washed out with PBS or TBS buffer. HRP substrate was added to visualize the bound antibodies. The HRP enzymatic reaction produced a blue product that changed to yellow after adding the acidic stop solution. The yellow absorbance was directly correlated to the amount of sample protein captured with the antibodies. Optical densities were determined with a microplate reader (Biorad Laboratories, Milan Italy) at 450 nm.

#### Zonulin 1 expression

We performed immunofluorescence staining to investigate ZO-1 expression in the tight junctions of human bladder cells (RT112). 3D model cultures were pretreated with TNF-α. Next, the cultures were washed with PBS, fixed with 4% w/v paraformaldehyde (Sigma Aldrich, Milan Italy), and permeabilized with 0.2% v/v Triton X-100 (Sigma Aldrich, Milan Italy) in PBS. Non-specific sites were blocked by incubating in PBS the contained 10% v/v bovine serum and 1% w/v bovine serum albumin. Next, we added a primary rabbit polyclonal anti-ZO-1 antibody (1: 300 v/v dilution in blocking buffer; Abcam, Cambridge, UK). Cells were incubated with primary antibodies overnight. Then, we added a secondary antibody (FITC-conjugated, anti-rabbit, 1:2000 dilution; Life Technologies, Italy) and incubated for 60 min. Next, cells were washed three times in PBS and mounted on microscope slides with ProLong Gold Antifade Mountant (Life Technologies, Italy), applied directly to the labeled cells. Nuclei were stained with 0.5 μg/mL Hoechst (2′-(4-hydroxyphenyl)-5-(4-methyl-1-piperazinyl)-2,5′-bi-1H-benzimidazole trihydrochloride hydrate, bisBenzimide; Sigma-Aldrich, Milan Italy). Fluorescence images were captured with a fluorescence microscopy system (Zeiss, Milan Italy), and images were elaborated with AxioVision 4.8.2.

### Statistical analyses

Differences between groups were evaluated with the student’s t test. Statistical significance was set to p<0.05. For all comparisons, we averaged the results of three independent experiments, each performed in duplicate, to avoid potential errors due to variations in cell cultures.

## Results

### Reagent analyses

#### Hydrodynamic characterization

We analyzed sterilized raw materials and the HA/CSb formulation with SEC-TDA (Table 1). These preparations were used in 2D and 3D biological assays.

#### Elemental analyses of commercial products

Results from the elemental analyses showed that IALURIL (lot 180505) contained 2270 ppm Ca^++^ and 2290 ppm Na^+^. INSTILLAMED LK04331 contained only 104 ppm Ca^++^ and 5394 ppm Na^+^.

### Experiments with the 2D model

#### Anti-inflammatory effect: IL-6 and IL-8 gene expression

**Fig 2:**
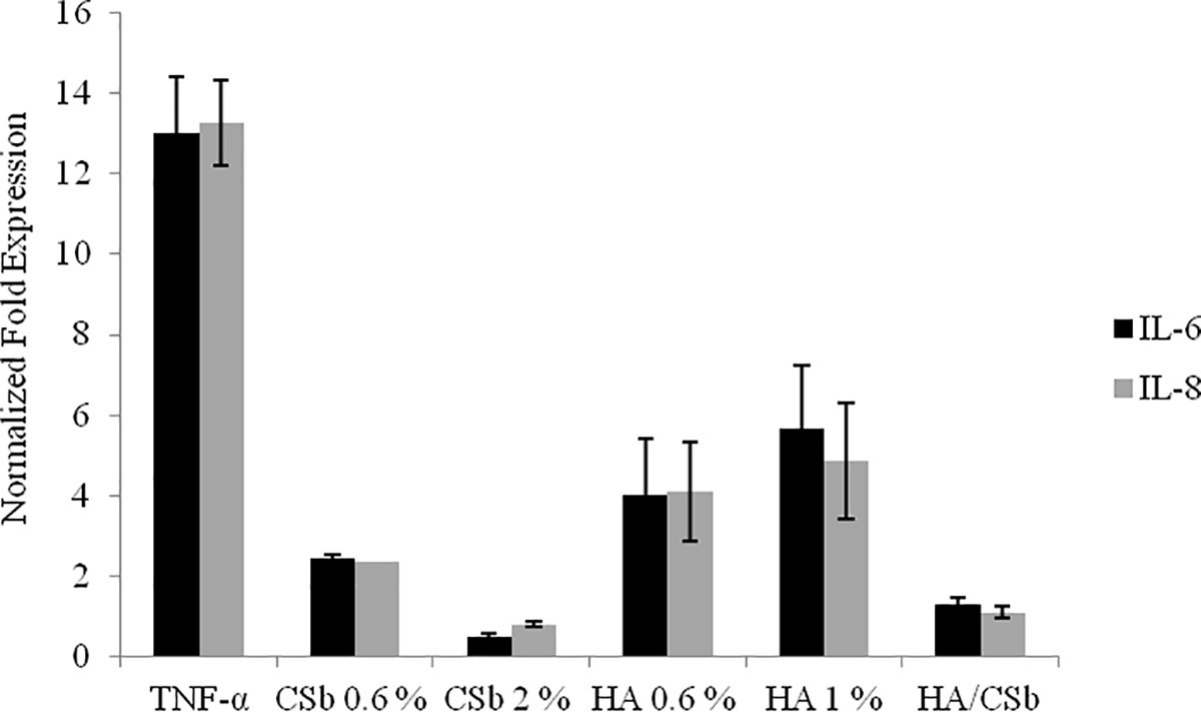
Representative GAG effects on IL-6 and IL-8 gene expression in the 2D bladder model after a TNF-α challenge. qRT-PCR results show the effects of 4-h treatments with CSb at 0.6% and 2% (w/v) concentrations, HA at 0.6% and 1% (w/v) concentrations, and a fixed HA/CSb formulation on bladder cells challenged with TNF-α. Data are the means ± SD of triplicate assays. *p<0.01 compared to TNF-α alone; §p<0.01 CSb compared to HA.

An intermediate treatment time (4h) was chosen to represent GAG effects on inflammation at an early stage. Two concentrations of CSb (0.6% and 2% w/v) and HA (0.6 and 1% w/v) significantly reduced both IL-6 and IL-8 gene expression. CSb was more effective than HA. The HA/CSb formulation also effectively reduced the harmful effect of TNF-α on bladder cells. Specifically, the HA/CSb formulation reduced IL-6 and IL-8 gene expression levels by about 8-fold, compared to TNF-α treatment alone (Fig 2).

#### NF-κB protein levels

**Fig 3:**
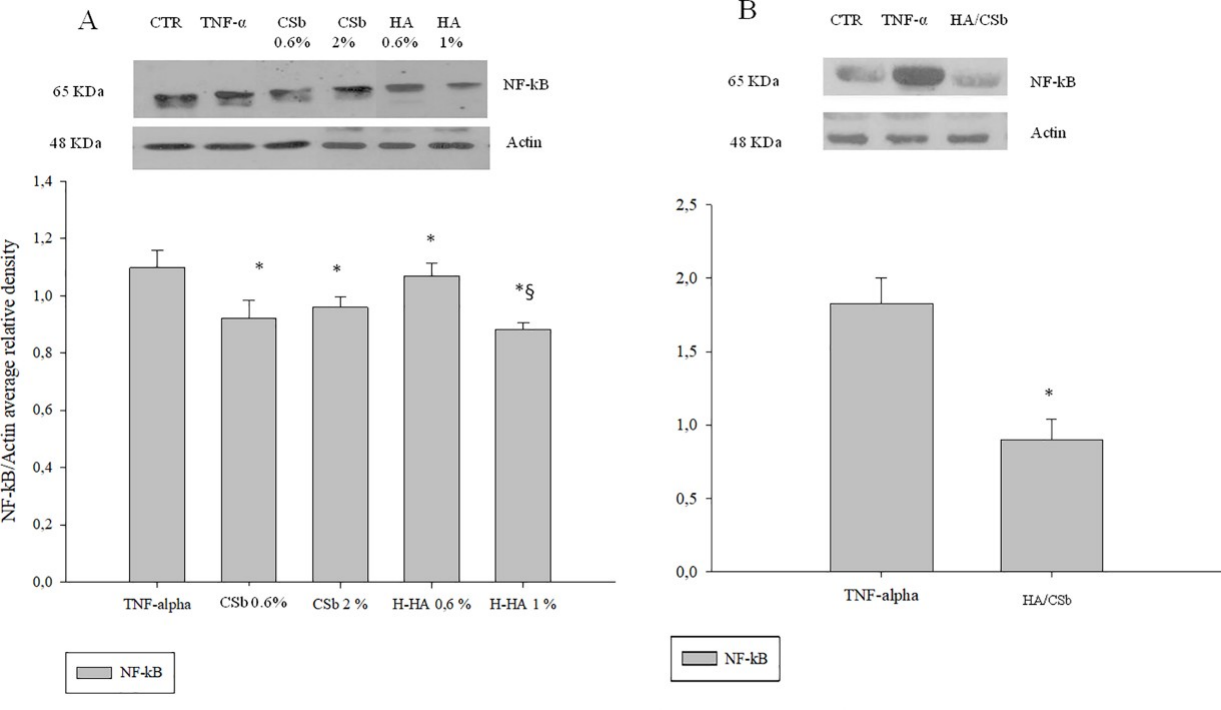
GAG effects on NF-κB expression in a 2D bladder model challenged with TNF-α. Western blot analyses show NF-κB expression in the presence of (A) single HA and CSb treatments and (B) the HA/CSb formulation. Data are the means ± SD. *p<0.01 compared to TNF-α alone; §p<0.01 for HA (1% w/v) compared to CSb (A).

We assessed the efficacy of CSb and HA on NF-κB protein modulation with western blot analyses (Fig 3). A low CSb (0.6% w/v) concentration efficiently reduced NF-κB expression The HA/CSb formulation also effectively reduced inflammation; the NF-κB protein level was reduced by 2-fold, compared to TNF-α treatment alone.

#### IL-6 and IL-8 protein levels

**Fig 4:**
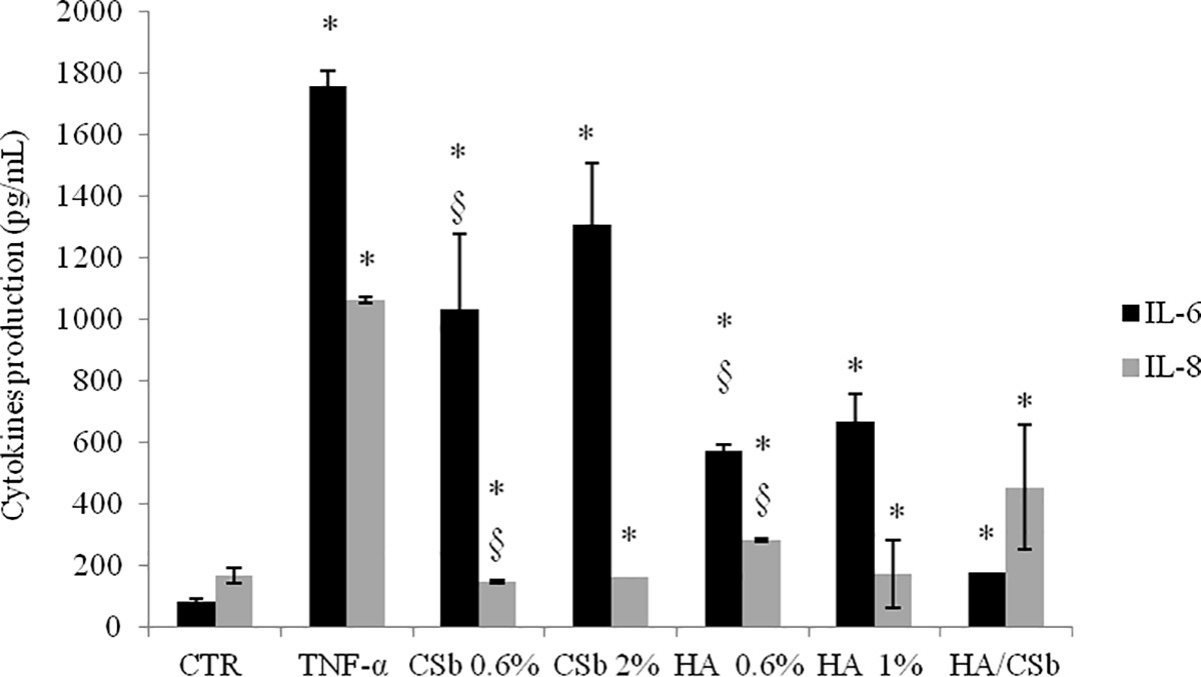
GAG effects on IL-6 and IL-8 protein expression in the 2D bladder model after a TNF-α challenge. ELISA results show the effects of CSb at 0.6% and 2% (w/v), HA at 0.6% and 1% (w/v), and a fixed HA/CSb formulation on bladder cells challenged with TNF-α. Data are the means ± SD. *p <0.01 compared to TNF-α treatment alone; §p<0.01 CSb 0.6% compared to 0.6% HA.

The HA treatment reduced IL-6 protein levels by 2.5 fold, and CSb treatment reduced IL-6 protein levels by 30–40%, compared to TNF-α alone (Fig 4). In addition, IL-8 protein levels were reduced up to 9-fold with CSb and by 6-fold with HA. The HA/CSb formulation efficiently reduced both IL-6 and IL-8 protein levels; this effect was greater on IL-6 (10-fold reduction) than on IL-8 (50% reduction), compared to TNF-α-treated cells (Fig 4).

#### Antibacterial activity: hBD-2 protein expression

**Fig 5:**
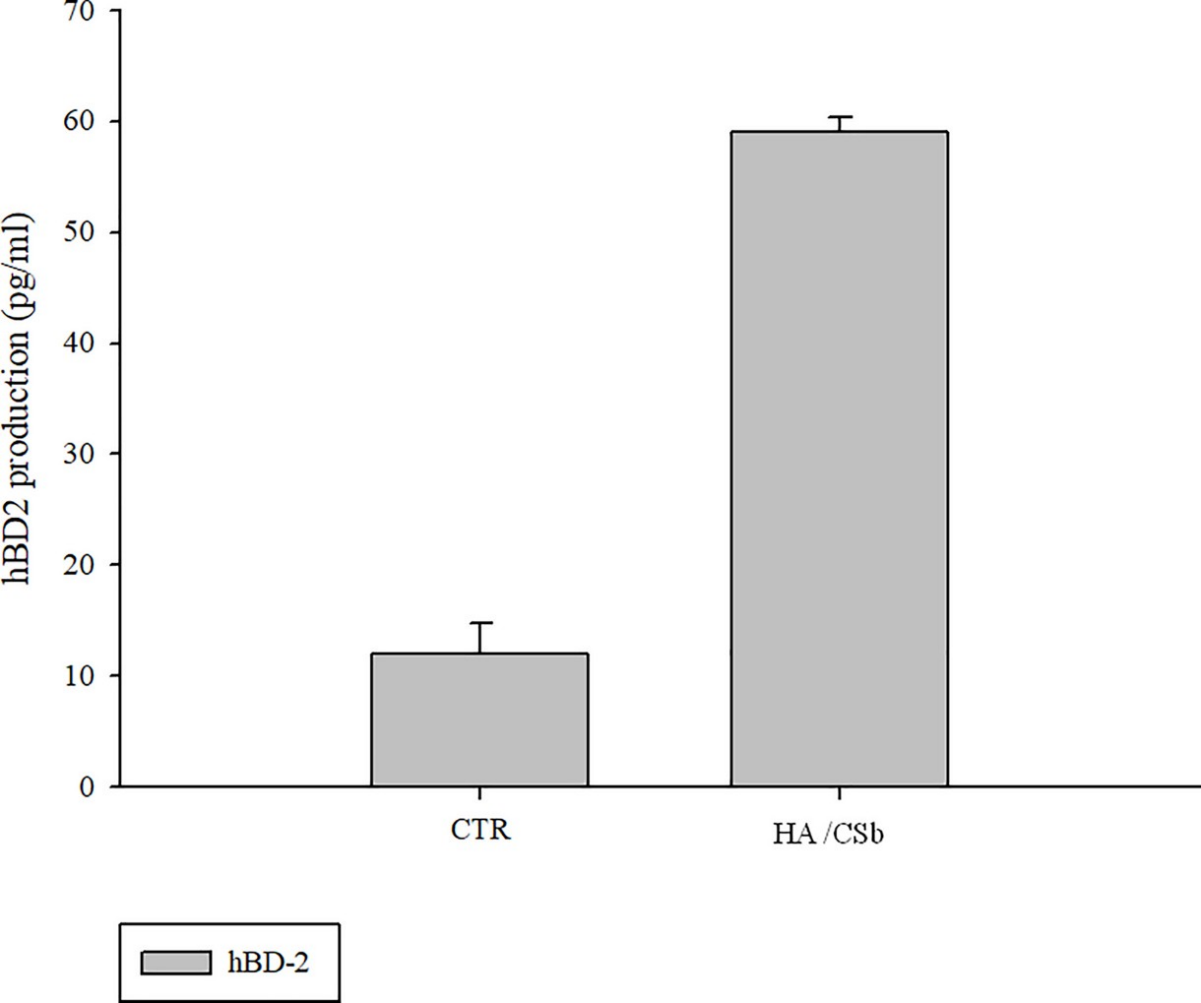
hBD-2 protein expression in epithelial bladder cells stimulated with GAGs. ELISA assays show hBD-2 expression after 72h of GAG treatment. Data are the means ± SD. *p<0.01 compared to control.

In bladder cells cultivated in standard medium, the addition of GAGs increased hBD-2 expression. This effect indicated that GAGs stimulated the biosynthesis of antibacterial peptides. In particular, incubation with HA/CSb increased the expression of defensin by >5-fold after 72h (Fig 5).

### Experiments with the 3D model

**Fig 6:**
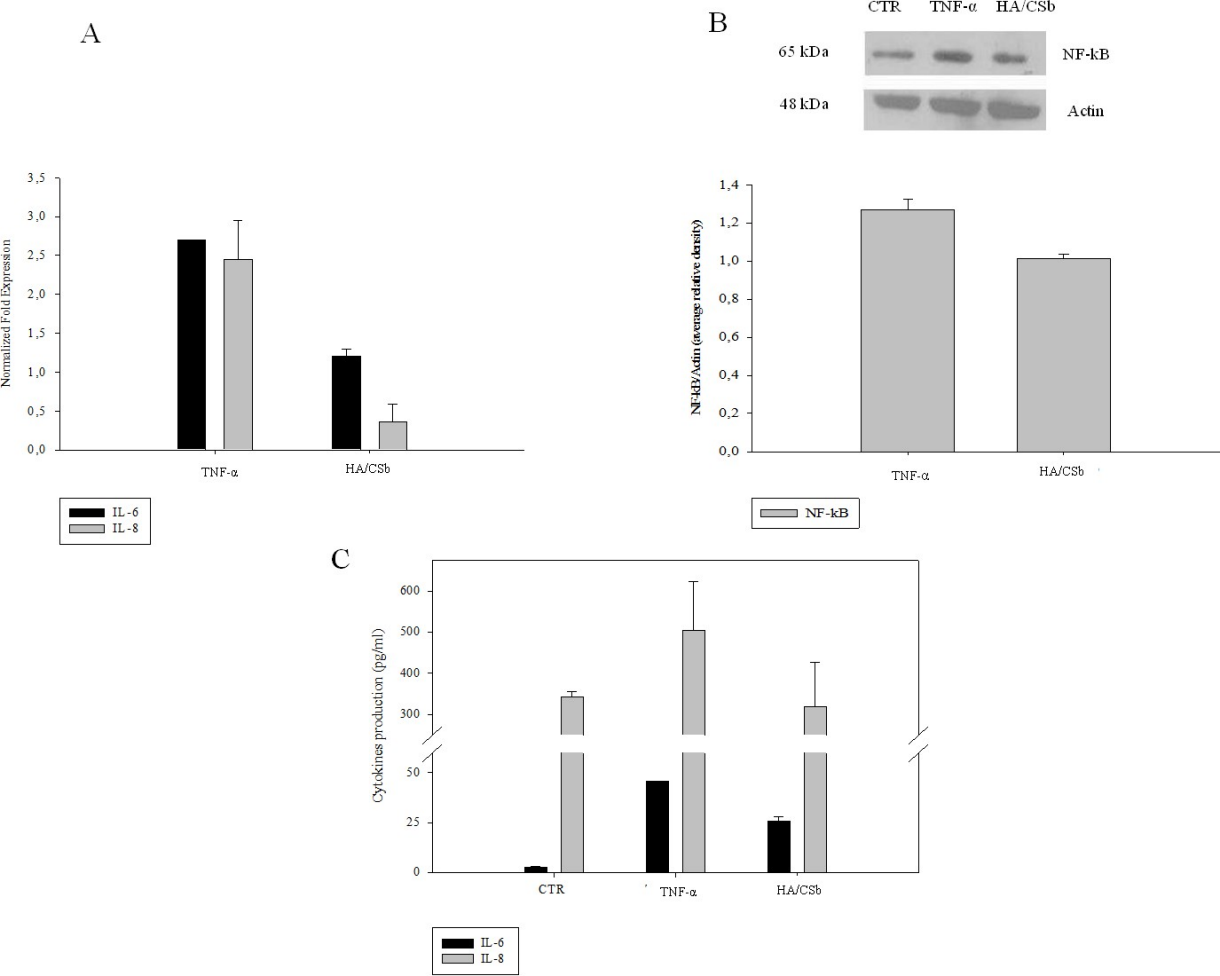
Effects of the HA/CSb formulation on a 3D bladder model challenged with TNF-α. (A) qRT-PCR results show HA/CSb effects on IL-6 and IL-8 gene expression. (B) Western blots show HA/CSb effects on NF-κB protein expression. (C) ELISA results show HA/CSb effects on IL-6 and IL-8 cytokine production after 48 h. Data are the means ± SD. *p<0.01 compared to TNF-α alone.

To corroborate the evaluations obtained with monolayers, was used the 3D bladder epithelium model to test the efficacy of the HA/CSb formulation. The anti-inflammatory activity of HA/CSb was assayed with qRT-PCR. This gene expression analyses showed that HA/CSb was equally efficient in reducing both IL-6 and IL-8 expression (Fig 6A). In addition, after 48 h of HA/CSb treatment, western blots showed that NF-κB expression levels were reduced (Fig 6B). Also, in agreement with gene expression results, the 3D model results confirmed that HA/CSb could efficiently reduce the protein levels of both cytokines (Fig 6C).

#### Immunofluorescence detection of Zonulin 1

**Fig 7:**
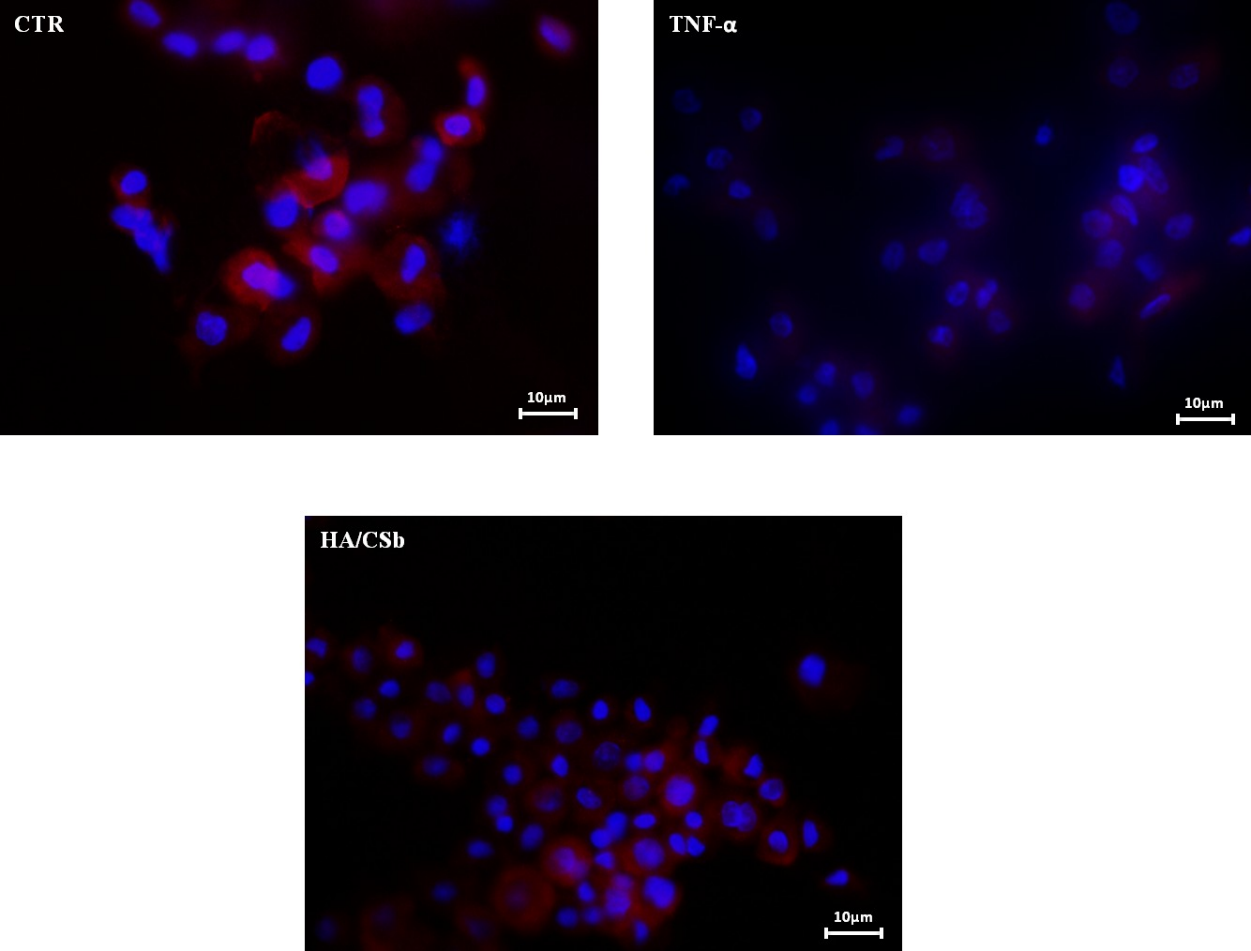
Effects of the HA/CSb formulation on ZO-1 expression in a human bladder 3D cell model challenged with TNF-α. Immunofluorescence image shows ZO-1 (red) expression after 48 h of HA/CSb treatment. Nuclei are shown in blue.

The expression of tight junction proteins influences cell-cell contact, and thus, the permeability of the bladder endothelium [6]. Therefore, in our IC/BPS model, we considered ZO-1, a representative protein for cell/cell contact. We evaluated ZO-1 expression with immunofluorescence staining [19]. Fig 7 shows that a TNF-α insult reduced ZO-1 expression, which damaged tissue integrity. The addition of HA/CSb re-established the expression of ZO-1 and induced cell proliferation. These activities returned the bladder tissues to a physiological status.

### Comparative evaluation of two medical devices on the market

#### Hydrodynamic characterization

The SEC-TDA hydrodynamic analyses showed that the HA in IALURIL had a higher molecular weight (245 ± 12 kDa) than INSTILLAMED (199 ± 2.50 kDa). The CS in the commercial devices show different peak profiles. The molecular weights of CS were 16.15 ± 0.20 kDa in IALURIL and 26.46 ± 0.20 kDa in INSTILLAMED.

#### Dynamic viscosity and mucoadhesive properties

**Fig 8:**
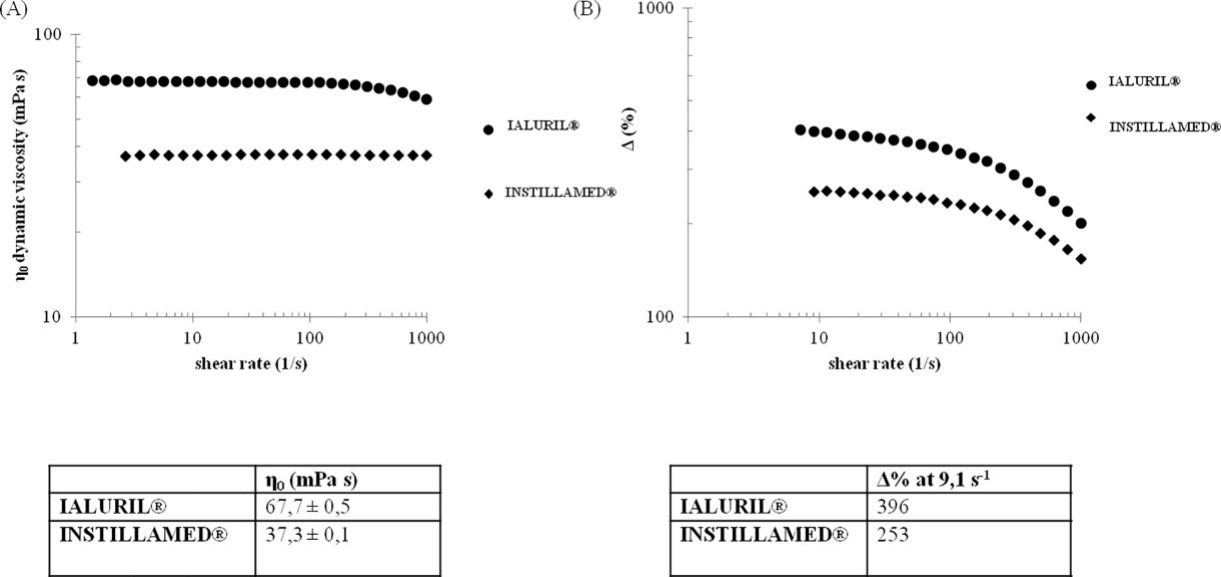
Comparison of two commercial medical devices for HA/CSb instillation. (A) Flow curves in the range of 1–1000 s^-1^ were performed at 37°C; η0 values are shown for IALURIL and INSTILLAMED. (B) The mucoadhesion indexes (Δ%) of IALURIL and INSTILLAMED is plotted as a function of the shear rate (a) and Δ% measured at a shear rate of 9.1 s^-^1 according to previously reported method [10].

The flow curves for IALURIL and INSTILLAMED are shown in Fig 8A. The two samples behaved differently: INSTILLAMED exhibited a viscosity that remained constant over the whole range of shear rates tested. In contrast, IALURIL exhibited a slight, but clear, shear thinning behavior at shear rate values above 100 s^-1^. Furthermore, INSTILLAMED had a 1.8-fold lower zero-shear viscosity than IALURIL. The mucoadhesion indexes for the two formulations, as a function of the shear rates, are shown in Fig 8B. For both formulations, the mucoadhesion index decreased with increases in the shear rate. IALURIL showed higher mucoadhesiveness than INSTILLAMED over the entire range of shear rates tested. In particular, at 9 s^-1^, IALURIL had a 1.6 fold higher mucoadhesion index than INSTILLAMED. All parameters were measured in triplicate and the standard deviation was ≤5%. Thus, the two mucoadhesive profiles were significantly different (Fig 8B).

#### Biological activity: IL 6 and IL 8 cytokine analyses

**Fig 9:**
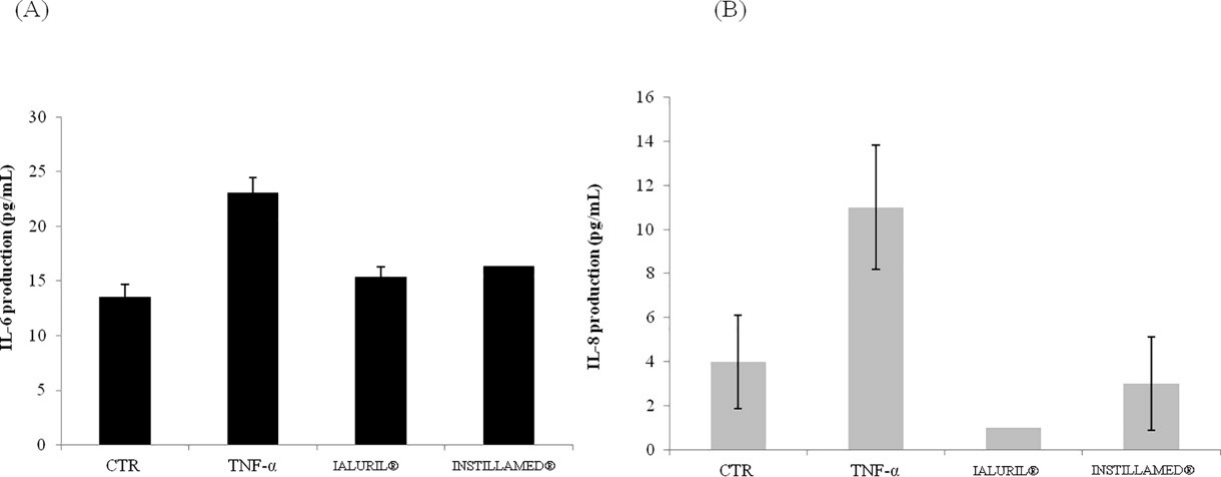
IALURIL and INSTILLAMED effects on cytokine expression in an inflammation model challenged with TNF-α. ELISA results show reductions in (A) IL-6 and (B) IL-8 protein expression with IALURIL and INSTILLAMED treatment compared to TNF-α alone.

IL-6 and IL-8 cytokine levels increased by >2-fold with TNF-α treatment, compared to control conditions. The addition of IALURIL reduced the IL-6 and IL-8 (Fig 9A and 9B) expression levels by 1.6 and 11-fold, respectively, compared to TNF-α challenged cells. Similarly, INSTILLAMED reduced the IL-6 and IL-8 expression levels by 1.6 and 4.4 fold, respectively. These results demonstrated that IALURIL was more efficient than INSTILLAMED as anti-inflammatory agent that modulated IL-6 and IL-8 expression.

## Discussion

Currently, HA and CS are the most commonly used GAGs for intravesical treatments. The combination of CS (2.0%w/v) and HA (1.6% w/v) is the latest available formulation for GAG replenishment therapy. Randomized controlled trials, clinical experience, and feedback from patient support groups have confirmed the beneficial effects of intravesical HA, alone or in combination with CS, for symptomatic relief from IC/BPS [4]. In the last ten years, clinical studies that investigated the HA/CS combination have shown significant beneficial effects in patients with IC/BPS [20, 21, 22]. Although intravesical instillation of exogenous GAGs was successful in patients with IC/BPS, we lack animal models that accurately reflect the disease that can be used to generate reliable non-clinical data. In fact, IC/BPS is particularly challenging to model, because it is a symptom-driven disorder of unknown etiology and pathophysiology. Due to patient heterogeneity and the multifactorial complexities of the disease, it is unlikely that any single animal model can reflect the complete human syndrome, as reported by the MAPP Research Network [23]. Due to this lack of validated animal models, in this study, we explored GAG therapy in 2D and 3D *in vitro* bladder urothelium models challenged with TNF-α, which resembled the inflammatory status of bladder cells in IC/BPS.

To investigate the anti-inflammatory effects of formulations based on HA (MW 500–1800 kDa) and CS (MW ≈20 kDa) [24], we evaluated specific biomarkers. As previously reported, we exposed cells to TNF-α, which mediated up-regulations of NF-κB, IL-6, and IL-8 [6, 25, 26]. These pro-inflammatory cytokines are known to be up-regulated in patients with IC/BPS [27, 28].

In addition to GAG layer replenishment, HA directly interacts with the cell surface. This interaction might reduce urothelium permeability. Moreover, because HA can bind to several cellular receptors [29], it can reduce cytokine secretion, and thus, the inflammatory status. In turn, these activities might alleviate IC/BPS symptoms [6]. When administered alone, CS was reported to play a key role in inflammation-based diseases (such as osteoarthritis). CS has been referred to as an antibacterial/bacteriostatic substance; therefore, it has been well assessed for use in pathologies that are generally associated with infections or dismicrobism. Instillations of 0.2% CS, given weekly for 4 weeks, then monthly, for 12 months, improved symptoms in 67% of patients [30]. Additionally, instillations of 2% CS improved symptoms in 60% of patients at 6 months [31]. Some have speculated that CS might stimulate proteoglycan synthesis, which could increase the production of intravesical HA, and thus, reconstitute the urothelium [32]. Alternatively, the biochemical mechanism underlying the improvement in pathology might involve the action of GAGs on the inflammation process. In that process, TNF-α stimulation activates the NF-κB protein [33], which moves from the cytoplasm to the nucleus, where it then induces the transcription of inflammatory cytokines, IL-6 and IL-8 [6] (Fig 1).

In the present study, we tested increasing concentrations of CSb and HA (from 0.6% to 2% w/v). In our experiments, CSb actively reduced inflammation markers, IL-6 and IL-8. Thus, in addition to the findings of Rooney and collaborators [6], who demonstrated that HA played a positive role *in vitro*, we showed that CSb also reduced cytokines when used alone. Moreover, we found that the HA/CSb formulation reduced NF-κB expression in TNF-α challenged bladder cells *in vitro*. In addition, HA/CSb had positive effects on both monolayers and bladder cells in a 3D model. Consistent with other studies [34], we showed that HA/CSb increased the expression of hBD-2 at both the gene and protein levels. Furthermore, in a 3D bladder model challenged with TNF-α, we confirmed the efficacy of HA/CSb on reducing the permeability of the bladder endothelium [6]. Specifically, the levels of the tight junction protein, ZO-1, increased with HA/CSb treatment, compared to TNF-α-treated cells. This effect renewed the tissue to a more physiological condition, with respect to tight junction/cell-cell binding.

Despite its obvious limitations, the *in vitro* approach permitted us to demonstrate that HA/CSb could improve the integrity and function of bladder epithelium. This experimental set up was based on the correlation between the IC/BPS pathogenesis and GAG-related disorders. Our findings supported the importance of using GAG-based formulations as therapies for these debilitating and often chronic diseases. The protocols we implemented permitted an *in vitro* comparison of biomarker levels, avoiding the heterogeneity of *in vivo* models. Considering the criticisms toward robustness of animal model, the model presented here might provide a valid platform for unraveling the biochemical mechanisms involved in improving bladder tissue functionality though GAG based formulations.

Finally, in the present study, we compared two commercial products, IALURIL and INSTILLAMED, on key features, specifically: *i*) the calcium and sodium ion concentrations; *ii*) the viscosity and mucoadhesive properties; and *iii*) the biological outcomes. In fact, it was suggested that Ca^++^ might play a role in the effectiveness of treatments for bladder disorders. Our analyses showed that IALURIL contained over 20-fold more divalent cations than INSTILLAMED, and INSTILLAMED mainly contained Na^+^ as the counterion for the anionic GAGs. In general, the higher the viscosity and mucoadhesion properties of a product, the better the expected performance. Our results (Fig 8) suggested that IALURIL might be retained longer in the bladder epithelium, and this might confer superior efficacy compared to INSTILLAMED.

The investigated flow parameters depended on the molecular weights, concentrations, and ionic strengths of GAGs. Moreover, the different types of ions might contribute to the diverse behaviors of these formulations. In fact, studies have shown that the Ca^++^ concentration, in addition to the molecular weight, might affect biopolymer chain behavior [35,10]. The hydrodynamic characterization of the two medical devices with SEC-TDA showed that they had different MW distributions, probably due to differences in the raw materials used by the companies and the different ionic strengths (which was higher in IALURIL). In addition, IALURIL and INSTILLAMED presented different biological features, despite the fact that both reduced pro-inflammatory cytokines. IALURIL was the more powerful of the two, due to its stronger reduction of IL-8. This difference in the two formulations might be due to differences in HA MWs, the different divalent cation concentrations, and/or the different CS concentrations (e.g., which might depend on the extractive source).

A potential limitation of the present study was that our findings were not supported by *in vivo* data. However, the 3D model of the human bladder urothelium used in this study was an appropriate *in vitro* model for testing the efficacy of GAG therapy in reducing the inflammatory response. This approach could represent an alternative to *in vivo* approaches, considering the lack of validated chronic cystitis animal models [20].

## Conclusions

We demonstrated that an appropriate *in vitro* model of IC/BPS was useful for comparative analyses of the efficiency of different therapeutic formulations in modulating inflammation at the molecular level and tissue integrity at the morphological level. Our results showed that the HA/CSb formulation had a positive anti-inflammatory effect. It improved cell-cell interactions that might contribute to the re-establishment of the bladder barrier. Our results suggested that the mechanism underlying the effects of HA/CSb involved the NF-κB pathway. Our findings might provide a scientific rationale for the use of GAG-based formulations.

We also compared two commercial medical devices that contained HA and CS in combination. We showed that they had different dynamic viscosity and mucoadhesive properties. Our results indicated that IALURIL contained more Ca^++^ ions and behaved with superior mucoadhesion compared to INSTILLAMED. Therefore, its ability to remain in contact with the bladder epithelium conferred superior efficacy compared to INSTILLAMED. Although both products reduced the cytokine levels in the *in vitro* IC/BPS model, IALURIL was more effective than INSTILLAMED in reducing IL-8 to more physiological levels.
